# Preface of the Special Issue “COVID-19 Infection and Hematological Malignancies”

**DOI:** 10.3390/cancers14184497

**Published:** 2022-09-16

**Authors:** Mingyi Chen, Larry D. Anderson

**Affiliations:** 1 Department of Pathology and Laboratory Medicine, Hematopathology, University of Texas, Southwestern Medical Center, Dallas, TX 75390, USA; 2 Myeloma, Waldenstrom’s, and Amyloidosis Program, Simmons Comprehensive Cancer Center, UT Southwestern Medical Center, Dallas, TX 75390, USA

Coronavirus disease 2019 (COVID-19), caused by severe acute respiratory syndrome coronavirus 2 (SARS-CoV-2), was declared a pandemic by the World Health Organization (WHO) in March 2020. COVID-19 has spread worldwide, causing several million deaths. Leukemia, lymphoma, and myeloma represent heterogeneous hematological malignancies (HMs), which are characterized by severe immunosuppression and leave patients at high risk of COVID-19 infection and developing severe and life-threatening complications [1,2,3]. A better understanding of the risk factors for adverse outcomes may facilitate improved clinical management of these patients.

In the past three years, many studies have focused on the incidence and clinical evolution of COVID-19 in patients with HMs [4,5,6,7]. The reason patients with HMs are particularly vulnerable to SARS-CoV-2 infection may also be related to the detrimental effects of anti-neoplastic regimens (chemotherapy, BTK or PI3 kinase inhibitors, monoclonal antibodies for CD20, CD30 or CD38 and CD19 CAR-T therapy, etc.) on the immune system [8,9,10,11]. More importantly, the long-term impact COVID19 infection in the clinical, translational, and basic research topics in HMs prevention, initiation, progression, and treatment capture the most seminal studies in the rapidly growing area of immunology, immunotherapy, and cancer microenvironment [12,13,14,15,16].

The high morbidity and mortality rates reported in patients with hematologic malignancies underscore the vulnerability of this patient population [6,7,8,9]. We are interested in studies that shed light on the epidemiology, risk factors, pathophysiology, and outcomes of COVID-19 infection among patients with HMs [1,2,3,4,5,6,7,8]. It is very encouraging to observe the benefit of COVID-19 vaccinations as a marked reduction in the risk of infections amongst HM patients [12,13,14,15,16]. Many studies proved the effective benefit of vaccinations in the protection of patients from COVID-19 infection, especially reduced the risk of COVID-19-related mortality in patients with HMs [17,18,19,20].

We are inviting papers on the topics listed below for submission to the Special Issue “*COVID-19 Infection and Hematological Malignancies*”. This series of articles are presented by an international team of experts in the fields of hematology, virology, pathology, immunology, and infectious disease. With their dedication, deep knowledge, and understanding of the pathophysiology of disease, participation in international studies and projects, all of these assets help to publish high-quality papers in the following topics.

1. Discussion of the risk of COVID-19 infection in patients with underlying hematological malignancies. The significance of appropriate prevention and vaccination for those high-risk patients. The screening of antibody tests after COVID infection and/or vaccination in patients with leukemia, lymphoma and myeloma.
2. COVID-19-infection-induced changes in hematological malignancies and coagulation manifestations as prognostic markers in the prediction of disease severity. Performing early triage and the timely initiation of effective management may prevent disease progression and reduce the overall mortality rate.
3. Improved understanding of COVID-19 epidemiology in patients with HMs (including hematopoietic stem cell transplant recipients). The results obtained will improve knowledge regarding the prevalence of this complication in the different categories of patients with HMs.
4. Outcomes of patients with hematologic malignancies and COVID-19, with special emphasis on systematic reviews and meta-analyses to estimate the risk of death and other important outcomes for these patients.
5. The treatment strategies for patients with highest-risk HMs with COVID-19 infection, and a summary of guidelines regarding clinical decisions for patients with hematological neoplasms in the COVID-19 pandemic.
6. Recognition of COVID-19-vaccine-induced lymphadenopathy as a diagnostic dilemma for radiologists and pathologists. The documentation of vaccination status is critical to decrease unnecessary biopsies and alleviate patient anxiety.

In summary, this Special Issue is dedicated to the diagnosis and treatment of hematological malignancies during the era of COVID-19. We thank all the authors who will contribute articles regarding these interesting and timely topics. We hope this special Issue is educational for caregivers in their daily practice toward enabling high-quality patient care.
